# miR-26a-5p Activates Antiviral Innate Immunity via Direct Binding and Activation of RIG-I

**DOI:** 10.3390/v18091005

**Published:** 2026-09-12

**Authors:** Jiasong Xiong, Xian Lin, Wei Tang, Lili Wu, Qin Chen, Mengke Li, Xindi Huang, Lianzhong Zhao, Shiyun Chen

**Affiliations:** 1 State Key Laboratory of Virology and Biosafety, Wuhan Institute of Virology, Chinese Academy of Sciences, Wuhan 430071, China; xiongjiason@163.com (J.X.);; 2 University of Chinese Academy of Sciences, Beijing 100049, China; 3 Hubei Jiangxia Laboratory, Wuhan 430071, China; 4 Biomedical Research Institute, Hubei University of Medicine, Shiyan 442000, China; 5 School of Basic Medical Science, Hubei University of Medicine, Shiyan 442000, China

**Keywords:** miR-26a-5p, RIG-I, ligand, non-canonical function, broad-spectrum antiviral activity

## Abstract

Emerging evidence has revealed the critical roles of microRNAs (miRNAs) in the regulation of innate immune responses. Nevertheless, it remains poorly understood whether specific miRNAs can directly modulate retinoic acid-inducible gene I (RIG-I), a principal sensor of cytoplasmic viral RNA and a key initiator of antiviral innate immunity. In this study, we identified miR-26a-5p as a potent activator of the innate antiviral immune responses. Notably, this activation operates independently of the canonical miRNA pathways of translational repression and mRNA degradation. Instead, we demonstrated that miR-26a-5p exerts its immunostimulatory effects by specifically binding to the RIG-I receptor. RNA immunoprecipitation and RNA pull-down assays confirmed a direct physical interaction between miR-26a-5p and the RIG-I protein. Subsequent site-directed mutagenesis verified that an AU-rich motif is critical for miR-26a-5p-driven RIG-I activation. Viral challenge experiments demonstrated that miR-26a-5p confers broad-spectrum, RIG-I-dependent antiviral activity against both DNA viruses (herpes simplex virus type 1 and Kaposi’s sarcoma-associated herpesvirus) and RNA viruses (influenza A virus and respiratory syncytial virus). Collectively, our study indicates that miR-26a-5p may serve as a ligand that binds to and activates the RIG-I receptor. This discovery uncovers a previously unrecognized mechanism through which host miRNAs regulate antiviral innate immunity.

## 1. Introduction

Viral infections caused by both DNA and RNA viruses pose a persistent threat to global public health. The innate immune system serves as the first line of host defense against viral infection, wherein pattern recognition receptors (PRRs) sense conserved pathogen-associated molecular patterns (PAMPs) to trigger antiviral responses. Among PRRs, RIG-I acts as a principal cytoplasmic sensor of viral RNAs, including 5′-triphosphate double-stranded RNA (5′ppp-dsRNA) derived from RNA viruses and transcripts generated during DNA virus infection [1,2]. Upon ligand binding, RIG-I undergoes conformational changes, oligomerizes, and interacts with the mitochondrial adaptor protein VISA (also known as MAVS, IPS-1, or Cardif) [3,4]. This cascade activates the transcription factors IRF3/7 and NF-κB, and drives the production of type I interferons, proinflammatory cytokines, and downstream interferon-stimulated genes (ISGs). Collectively, these events are indispensable for restricting viral replication and eliminating virus-infected cells [5].

A core feature underlying this protective activity is the ability of RIG-I to distinguish self-RNA from non-self-RNA. Since RIG-I was first identified, extensive research has sought to define the molecular and structural determinants of the immunostimulatory RNAs that activate it. These determinants include a 5′-terminal triphosphate (5′-ppp) or uncapped diphosphate (5′-pp) group, an RNA duplex structure, and an unmethylated 2′-O position on the 5′-terminal nucleotide [6,7,8]. As these characteristics are prevalent in viral RNAs yet scarce in host cytoplasmic RNAs, viral RNAs were long considered the primary physiological triggers of RIG-I activation [9]. Nevertheless, recent studies have demonstrated that several host cellular RNAs can also mediate RIG-I activation during viral infection. For instance, host 5S ribosomal RNA pseudogene 141 (RNA5SP141) directly binds and activates RIG-I during herpes simplex virus type 1 (HSV-1) infection [10]. Likewise, the RNase-cleaved internal transcribed spacer 2 (ITS2) region of 5S ribosomal RNA interacts with and activates RIG-I [11]. During the lytic infection of Kaposi’s sarcoma-associated herpesvirus (KSHV), downregulation of dual-specificity phosphatase 11 (DUSP11) enables host vault RNAs (vtRNAs) to bind and activate RIG-I [12]. These findings collectively establish that multiple classes of host-encoded endogenous RNAs can initiate RIG-I-dependent antiviral immunity, raising the question of whether additional non-coding RNA species serve similar roles.

Among the diverse classes of host non-coding RNAs, miRNAs stand out owing to their widespread cellular expression and multifaceted biological roles. miRNAs are small non-coding regulatory RNAs ranging from 21 to 25 nucleotides (nt) in length [13]. Ever since their initial discovery in *Caenorhabditis elegans* in 1993 [14,15], studies have uncovered their diverse molecular mechanisms of action [16,17,18]. The canonical function of miRNAs is to serve as “fine-tuners” of post-transcriptional gene regulation, exerting their effects by specifically binding to target mRNAs within the miRNA-induced silencing complex (miRISC) to repress translation or promote mRNA degradation [19,20]. A single miRNA can modulate hundreds of mRNAs in vivo, while an individual transcript may be targeted simultaneously by multiple miRNAs. This complex regulatory network allows miRNAs to participate in nearly every biological process, including cell cycle regulation, growth and development, tumorigenesis, virus infection, and immune system modulation [21,22,23,24]. Although numerous studies have demonstrated that miRNAs modulate RLR signaling via their canonical functions [25], a non-canonical function has also been reported for miR-136, which serves as an endogenous ligand that directly binds to and activates RIG-I [26]. However, such ligand-like functions of miRNAs remain poorly characterized and warrant further investigation.

To systematically identify miRNAs that function as endogenous RIG-I ligands, we performed RNA immunoprecipitation coupled with high-throughput sequencing (RIP-seq) with RIG-I as bait. Among the miRNAs enriched in RIG-I immunoprecipitates, miR-26a-5p stood out for its potent induction of type I interferon. Further biochemical analysis revealed that miR-26a-5p directly binds to RIG-I and colocalizes with the receptor, triggering receptor activation and stimulating type I interferon production independently of its canonical post-transcriptional function. Mutagenesis analysis identified that the AU-rich motif is critical for miR-26a-5p-triggered RIG-I activation. Collectively, these findings establish miR-26a-5p as a RIG-I ligand, capable of eliciting robust interferon responses and conferring broad-spectrum antiviral effects against both RNA and DNA viruses.

## 2. Materials and Methods

### 2.1. Cells and Viruses

All cells were cultured at 37 °C in a 5% CO_2_ humidified incubator. The HEK293T (CRL-3216), RAW264.7 (TIB-71), A549 (CCL-185), MDCK (CCL-34), HEp-2 (CCL-23), Hela (CCL-2), and Vero cells (CCL-81) (HEK293T: CRL-3216 RAW264.7: TIB-71 A549: CCL-185 MDCK: CCL-34 HEp2: CCL-23 HeLa: CCL-2 Vero: CCL-81) were purchased from the American Type Culture Collection (Manassas, VA, USA) and cultured in Dulbecco’s Modified Eagle Medium (DMEM) (C11995500BT, Thermo Fisher Scientific, Waltham, MA, USA) supplemented with 10% fetal bovine serum (FBS) (10099141, Thermo Fisher Scientific, Waltham, MA, USA), 100 U/mL penicillin, and 100 µg/mL streptomycin (C0222, Beyotime Biotechnology, Shanghai, China). RIG-I-knockout A549 cells and MDA5-knockout A549 cells were provided by Dr. Yan-Yi Wang and KSHV-GFP was provided by Dr. Jungang Chen. The influenza A virus (IAV) strain A/Puerto Rico/8/1934 (H1N1, PR8), HSV-1, and respiratory syncytial virus (RSV) were stored in our laboratory.

### 2.2. Antibodies and Reagents

Anti-p-TBK1 (ab109272) and anti-respiratory syncytial virus (ab20745) antibodies were purchased from Abcam (Cambridge, UK). Anti-p-IRF3 (4947) antibody was purchased from Cell Signaling Technology (Danvers, MA, USA). Anti-TBK1 (28397-1-AP), anti-IRF3 (11312-1-AP), anti-HA (66006-2-Ig), anti-Flag (20543-1-AP), anti-RIG-I (20566-1-AP), anti-MDA5 (21775-1-AP), rabbit IgG control (30000-0-AP), anti-GAPDH (60004-1-Ig), and CoraLite594-conjugated goat anti-mouse IgG (H + L) (SA00013-3) antibodies were purchased from Proteintech Group, Inc. (Rosemont, IL, USA). Poly(I:C) (HY-107202), puromycin (HY-K1057), chloroquine (HY-17589A), streptavidin magnetic beads (HY-K0208), protein A/G magnetic beads (HY-K0202), the type-I interferon receptor subunit 1 (IFNAR1)-blocking antibody Anifrolumab (HY-P99168), and human IgG1 isotype-control antibody (HY-P991631) were obtained from MedChemExpress LLC (Monmouth Junction, NJ, USA). HRP-labeled goat anti-rabbit IgG (H + L) (A0208) and HRP-labeled goat anti-mouse IgG (H + L) (A0216) antibodies were purchased from Beyotime Biotechnology (Shanghai, China).

### 2.3. miRNA Mimics, Small Interfering RNAs, and Plasmids

miR-26a-5p mimic (miR10000082-1-5), mimic control (miR1N0000001-1-5), single-stranded mimic of miR-26a-5p, 2′-O-methyl-modified miR-26a-5p mimic, and multiple mutant miR-26a-5p mimics were purchased from Guangzhou RiboBio Co., Ltd. (Guangzhou, China).

TLR3 small interfering RNAs (siTLR3), RIG-I small interfering RNAs (siRIG-I), and a negative control were purchased from Sangon Biotech (Shanghai, China). All small interfering RNAs sequences are listed below:

siTLR3-1 (Forward): CCUCUUCGUAACUUGACCAUU(dT)(dT),

siTLR3-1 (Reverse): AAUGGUCAAGUUACGAAGAGG(dT)(dT);

siTLR3-2 (Forward): CCAGCACAAUGAGCUAUCU(dT)(dT),

siTLR3-2 (Reverse): AGAUAGCUCAUUGUGCUGG(dT)(dT);

siTLR3-3 (Forward): GCCACCUAGAAGUACUUGA(dT)(dT),

siTLR3-3 (Reverse): UCAAGUACUUCUAGGUGGC(dT)(dT);

siRIG-I-1 (Forward): GAAGAUCUUGAGGAUAAGA(dT)(dT),

siRIG-I-1 (Reverse): UCUUAUCCUCAAGAUCUUC(dT)(dT);

siRIG-I-2 (Forward): GUAAUGCUGGUGUAAUUGA(dT)(dT),

siRIG-I-2 (Reverse): UCAAUUACACCAGCAUUAC(dT)(dT);

siRIG-I-3 (Forward): CAGAUGAAGCCUUGGAUUA(dT)(dT),

siRIG-I-3 (Reverse): UAAUCCAAGGCUUCAUCUG(dT)(dT).

Plasmids including Flag-MDA5, Flag-RIG-I, HA-RIG-I, IFN-β-Luc, and pRL-TK were purchased from Beyotime Biotechnology (Shanghai, China). Plasmids, miRNA mimics, and poly(I:C) were transfected into cells using Lipofectamine 3000 (L3000015, Thermo Fisher Scientific, Waltham, MA, USA). In all transfection experiments, miRNA mimics and their corresponding negative controls were transfected into cells at a final concentration of 60 nM, whereas siRNAs and their negative controls were transfected at a final concentration of 50 nM.

### 2.4. RNA Isolation and RT-qPCR Assay

Total cellular RNA was extracted from cells or immunoprecipitates using TRIzol reagent (15596018CN, Thermo Fisher Scientific, Waltham, MA, USA) according to the manufacturer’s instructions. Total RNA was reverse-transcribed into complementary DNA (cDNA) using the HiScript IV RT SuperMix for qPCR kit (R423-01, Nanjing Vazyme Biotech Co., Ltd., Nanjing, China), following the manufacturer’s recommended protocol. Quantitative PCR reactions were performed using Bio-Rad iTaq Universal SYBR Green Supermix (1725124, Bio-Rad Laboratories, Inc., Hercules, CA, USA) with the CFX96 Touch Real-Time PCR Detection System (1845097, Bio-Rad Laboratories, Inc., Hercules, CA, USA). The relative mRNA levels of *IFNA*, *IFNB1*, *ISG56*, *CCL5*, *CXCL10*, *IL6*, *Viperin*, *RIG-I*, *TLR3*, and *ISG15* were normalized to *ACTB* mRNA and analyzed by the comparative threshold cycle calculation method (2^−ΔΔCt^). The sequences of RT-qPCR primers used in this study are listed in Supplementary Table S1.

### 2.5. Stem-Loop RT-qPCR for miRNA Quantification

The expression of mature miR-26a-5p was quantified using stem-loop RT-qPCR as previously described [27]. Briefly, 1 μg total RNA extracted from cells was reverse-transcribed into cDNA using the HiScript IV 1st Strand cDNA Synthesis Kit (R412-01, Nanjing Vazyme Biotech Co., Ltd., Nanjing, China) with miR-26a-5p-specific stem-loop reverse transcription primers or U6 snRNA-specific reverse transcription primers in parallel, according to the manufacturer’s instructions. Quantitative PCR reactions were performed using Bio-Rad iTaq Universal SYBR Green Supermix with miR-26a-5p-specific qPCR primers or U6 snRNA-specific qPCR primers, following the manufacturer’s instructions.

Relative expression levels of miR-26a-5p were determined via the 2^−ΔΔCt^ method with U6 snRNA as the endogenous reference, and normalized to control samples. The reverse-transcription and qPCR primer sequences for the detection of miR-26a-5p and U6 snRNA are listed in Supplementary Table S1.

### 2.6. Dual-Luciferase Reporter Assay

HEK293T cells were seeded in 12-well plates and co-transfected with 0.5 μg of Flag-MDA5 or HA-RIG-I overexpression plasmids, 0.2 μg of IFN-β luciferase reporter plasmid, 10 ng of pRL-TK Renilla luciferase plasmid (internal control), and either miNC or miR-26a-5p mimic (final concentration, 60 nM). At 24 h post-transfection, cells were lysed, and luciferase activity was measured using the Dual Luciferase Reporter Assay Kit (DL101-01, Nanjing Vazyme Biotech Co., Ltd., Nanjing, China) according to the manufacturer’s protocol. Data were normalized for transfection efficiency by dividing firefly luciferase activity by Renilla luciferase activity.

### 2.7. RIP-qPCR and RIP-seq

HEK293T cells were seeded in two 10-cm culture dishes, and 15 μg of HA-RIG-I or Flag-MDA5 overexpression plasmids were transfected into the cells, followed by transfection with the miR-26a-5p mimic (final concentration, 60 nM) at 24 h post-transfection. After another 12 h of transfection, the cells were harvested and lysed using RIP lysis buffer for 30 min at 4 °C [28]. Cell lysates were centrifuged at 12,000× *g* for 10 min at 4 °C, and the supernatant was transferred to a new Eppendorf tube. Ten percent of the supernatant was saved as the input sample, and the remainder was subjected to RIP analysis. Supernatants from cells expressing HA-RIG-I or Flag-MDA5 were each divided equally into two aliquots. For the HA-RIG-I samples, 5 μg of anti-HA antibody or an isotype-matched IgG control was added to the respective aliquot; for the Flag-MDA5 samples, 5 μg of anti-Flag antibody or the corresponding isotype-matched IgG control was added, followed by overnight incubation at 4 °C. Protein A/G magnetic beads (30 μL) were then added to each sample, and the mixture was incubated for a further 4 h at 4 °C. Then, the beads were washed four times with RIP wash buffer and subsequently resuspended in DNase digestion buffer supplemented with 100 U RNase inhibitor (R0102, Beyotime Biotechnology, Shanghai, China) and 2 U DNase I (M0570S, New England Biolabs, Inc., Ipswich, MA, USA) for 30 min at 37 °C [28]. Beads were then sequentially washed four times and resuspended in 100 μL of RIP wash buffer, and 10% of this resuspended sample was saved for immunoblot analysis. Samples were subsequently treated with 4 U proteinase K (ST535, Beyotime Biotechnology, Shanghai, China) at 55 °C for 30 min to digest proteins and release RNA. Input and immunoprecipitated RNAs were extracted using TRIzol reagent, and the total RNA was subjected to RT-qPCR analysis.

The RIP-seq assay using RIG-I as bait was performed following a protocol similar to that described above. Total RNA was extracted from the co-purified Flag-RIG-I complexes immunoprecipitated with the anti-Flag antibody and subjected to next-generation sequencing (NGS) analysis.

### 2.8. RNA Pull-Down Assay

HEK293T cells were seeded in two 10-cm culture dishes, and 15 μg of HA-RIG-I or Flag-MDA5 overexpression plasmids were transfected into the cells, and 24 h post-transfection, the cells were lysed in RIP lysis buffer on ice for 30 min, followed by centrifugation at 12,000× *g* and 4 °C for 10 min. The resulting supernatant was then transferred to a new Eppendorf tube. Ten percent of the supernatant was saved as the input sample, while the remaining portion was subjected to subsequent RNA pull-down assays. Biotin-labeled miR-26a-5p, miR136, and miNC (10 μg each) were individually incubated with streptavidin magnetic beads (30 μL each) at 37 °C for 2 h. Then, the beads were washed three times with RIP lysis buffer to remove unbound miRNAs. The cell lysate supernatant was incubated with the miRNA-bound beads at 4 °C overnight. Subsequently, the beads were washed four times with RIP wash buffer to eliminate non-specific bindings. Both the input samples and the immunoprecipitated proteins were mixed with SDS loading buffer, boiled at 100 °C for 10 min to denature proteins, and then analyzed by Western blot analysis.

### 2.9. ELISA

A549 cells were seeded into 12-well plates. miNC and miR-26a-5p mimic were transfected into A549 cells at a final concentration of 60 nM. At 36 h post-transfection, the cell supernatant was collected, and the levels of type I interferons (IFN-α and IFN-β) were assayed using the Human IFN-α ELISA Kit (PI505, Beyotime Biotechnology, Shanghai, China) and the Human IFN-β ELISA Kit (PI572, Beyotime Biotechnology, Shanghai, China) according to the manufacturer’s instructions.

### 2.10. Western Blot

The protein samples from cell lysates or immunoprecipitates were mixed with SDS loading buffer (containing 5% β-mercaptoethanol), boiled at 100 °C for 10 min, and separated by 10% SDS-PAGE. Proteins were transferred onto 0.22 μm polyvinylidene difluoride (PVDF) membranes (ISEQ00010, Merck KGaA, Darmstadt, Germany) via wet transfer at 300 mA for 90 min under ice-cold conditions. Membranes were blocked with 5% non-fat milk or BSA in TBST for 1 h at room temperature, then incubated overnight at 4 °C with primary antibodies. After washing three times with TBST, membranes were incubated with HRP-conjugated secondary antibodies. Following three additional washes with TBST, protein bands were visualized with ECL reagent (E423-01, Nanjing Vazyme Biotech Co., Ltd., Nanjing, China) using a chemiluminescence imaging system (BG-gdsAUTO 710 MINI, Beijing Baygene Biotech Co., Ltd., Beijing, China).

### 2.11. Indirect Immunofluorescence Assay

A549 cells were seeded into confocal dishes and co-transfected with 0.2 μg of the HA-RIG-I overexpression plasmid along with either FAM-modified miNC or FAM-modified miR-26a-5p mimic (final concentration, 60 nM). At 24 h post-transfection, after two washes with PBS, the cells were fixed with 4% paraformaldehyde (PFA) for 30 min and permeabilized with 0.1% Triton X-100 in PBS for 15 min at room temperature. Following permeabilization, the cells were blocked with PBST containing 5% BSA at room temperature for 1 h, and then incubated with the indicated primary antibody at 4 °C overnight. After washing three times with TBST, the cells were incubated with the corresponding CoraLite594-conjugated secondary antibody for 1 h at room temperature. Following three additional washes with TBST, the nuclei were stained with DAPI (C1002, Beyotime Biotechnology, Shanghai, China) for 15 min at room temperature. Subsequently, confocal imaging analysis was performed using a Leica confocal laser scanning microscope (STELLARIS 8, Leica Microsystems GmbH, Wetzlar, Germany) with consistent imaging parameters.

### 2.12. Viral Infection Assay and Titer Measurement

Viral challenge assays with IAV and HSV-1 were performed in A549-WT, A549 MDA5-KO, and A549 RIG-I-KO cells, whereas RSV and KSHV-GFP were tested in A549-WT and A549 RIG-I-KO cells. Specifically, A549-WT, A549 MDA5-KO, and A549 RIG-I-KO cells were transfected with miNC or miR-26a-5p mimic at a final concentration of 60 nM. Twenty-four hours post-transfection, the cells were subjected to viral infection assays. For the IAV infection assay, the transfected cells were infected with IAV at a multiplicity of infection (MOI) of 0.01. The virus-containing cell supernatants were collected separately at the indicated time points post-infection. These supernatants were then used to determine viral titers in MDCK cells in the presence of TPCK-treated trypsin at a final concentration of 2 μg/mL. RSV was used to infect the transfected cells at an MOI of 0.1. Subsequently, RSV titers were determined via indirect immunofluorescence assay (IFA) in HEp-2 cells using anti-respiratory syncytial virus antibody. The transfected cells were infected with KSHV-GFP at an MOI of 1; KSHV replication was assessed by quantifying the GFP expression level. The transfected cells were infected with HSV-1 at an MOI of 0.1. Subsequently, HSV-1 titers in the collected cell supernatants were determined via plaque assay in Vero cells.

### 2.13. Type-I Interferon Receptor Blockade Assay

To determine whether Anifrolumab-mediated blockade of IFNAR1 could suppress miR-26a-5p-triggered type-I IFN signaling, A549 cells were pre-incubated with 20 μg/mL Anifrolumab or isotype-matched human IgG1 control antibody for 2 h. Cells were then transfected with miNC or miR-26a-5p mimic (final concentration, 60 nM). Twenty-four hours after transfection, ISG15 mRNA expression was quantified via RT-qPCR.

To investigate whether miR-26a-5p-mediated antiviral effects depended on the type-I interferon signaling pathway induced by miR-26a-5p-mediated RIG-I activation, A549 cells were pre-incubated with Anifrolumab or an IgG1 isotype control antibody for 2 h (20 μg/mL). Subsequently, cells were transfected with miNC or miR-26a-5p mimic at a final concentration of 60 nM. At 24 h post-transfection, the cells were infected with IAV (PR8 strain) at an MOI of 0.01. Cell-culture supernatants were harvested at 24 h post-infection for viral titer determination.

### 2.14. Statistical Analysis

All statistical analyses were performed using GraphPad Prism (v8.3.0, GraphPad Software, LLC, Boston, MA, USA). Statistical significance was determined by Student’s *t*-test. Differences with *p* < 0.05 were considered statistically significant: * *p* < 0.05, ** *p* < 0.01, *** *p* < 0.001, **** *p* < 0.0001; “ns” denotes no significance.

## 3. Results

### 3.1. miR-26a-5p Triggers the Production of Type I Interferons

**Figure 1:**
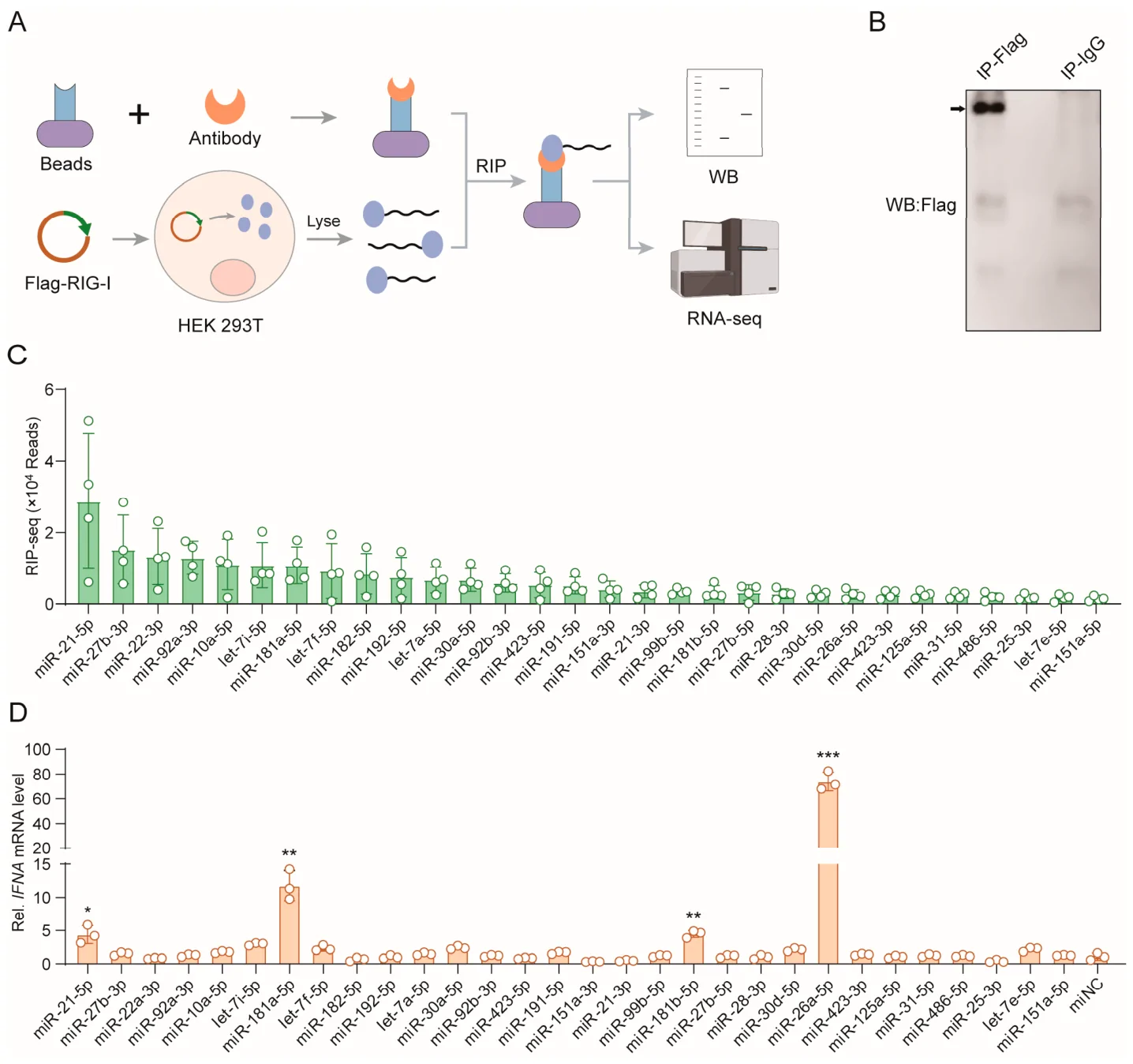
Screening and identification of RIG-I-binding miRNAs involved in the regulation of innate immunity. (**A**) Schematic workflow for identifying Flag-RIG-I-binding miRNAs in HEK293T cells via RIP-seq (Partially created in BioRender. https://BioRender.com/4vermk0). (**B**) Western blot validation of specific Flag-RIG-I enrichment by the anti-Flag antibody compared with the IgG isotype control in the RIP assays. The black arrow marks the specific RIG-I band. (**C**) Top 30 Flag-RIG-I-enriched miRNAs, ranked by normalized sequencing read counts. (**D**) RT-qPCR analysis of *IFNA* mRNA levels in A549 cells at 24 h post-transfection with mimics of the top 30 enriched miRNAs (final concentration, 60 nM). Statistical significance was determined by Student’s *t*-test. * *p* < 0.05, ** *p* < 0.01, *** *p* < 0.001.

To systematically identify miRNAs that directly activate RIG-I and modulate innate immunity, we performed RIP-seq (Figure 1A). We first validated the anti-Flag antibody for RIP and optimized the RIP protocol using HEK293T cells transiently transfected with Flag-RIG-I expression plasmid. RIP assays were performed using IgG and anti-Flag antibodies, respectively, and a specific blot band was detected in the anti-Flag immunoprecipitates (Figure 1B). RNA extracted from the antibody pull-down complexes was subjected to NGS analysis. Among the numerous miRNAs co-precipitated with RIG-I (Supplementary Table S2), including the previously reported miR-136 [26], the top 30 enriched miRNAs were ranked based on sequencing read counts (Figure 1C). To screen for candidate miRNAs capable of activating RIG-I, we synthesized mimics of these 30 miRNAs and individually transfected them into A549 cells, followed by RT-qPCR analysis of *IFNA* expression. Among them, miR-21-5p, miR-181a-5p, miR-181b-5p, and miR-26a-5p robustly induced type I interferon (*IFNA*) production (Figure 1D). Notably, miR-26a-5p displayed the strongest stimulatory activity and was therefore chosen as the primary focus for further mechanistic investigations.

**Figure 2:**
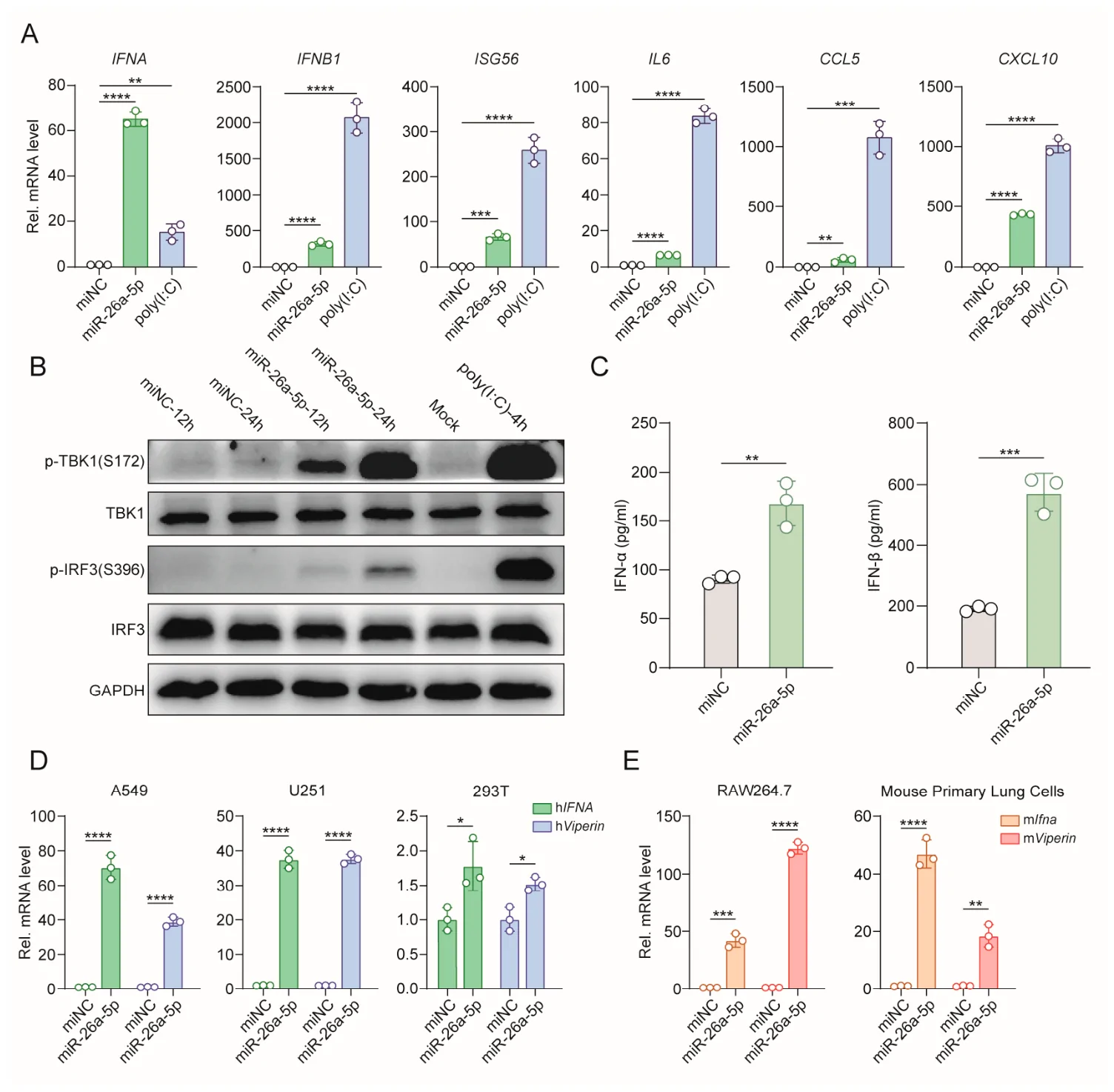
miR-26a-5p activates the type I interferon signaling pathway. (**A**) RT-qPCR analysis of *IFNA*, *IFNB1*, *ISG56*, *IL6*, *CCL5*, and *CXCL10* expression in A549 cells 24 h post-transfection with the miR-26a-5p mimic. miNC served as the negative control, and poly(I:C) served as the positive control. (**B**) Western blot analysis of p-TBK1 and p-IRF3 in A549 cells harvested at 12 h and 24 h post-transfection with miNC or miR-26a-5p mimic. Poly(I:C) transfection for 4 h served as the positive control. (**C**) ELISA analysis of secreted protein levels of IFN-α and IFN-β in the supernatants of A549 cells at 36 h post-transfection with miNC or miR-26a-5p mimic. (**D**,**E**) RT-qPCR analysis of *IFNA* and *Viperin* mRNA expression in human cells (A549, U251, and HEK293T) and murine cells (RAW 264.7 and mouse primary lung cells) at 24 h post-transfection with miNC or miR-26a-5p mimic. miR-26a-5p mimic and its negative control were transfected at a final concentration of 60 nM, and poly(I:C) at 1 μg/mL. Statistical significance was assessed by Student’s *t*-test. * *p* < 0.05, ** *p* < 0.01, *** *p* < 0.001, **** *p* < 0.0001.

To further confirm whether miR-26a-5p activates type I interferon production, we transfected the miR-26a-5p mimic into A549 cells, with poly(I:C) as a positive control. As shown in Figure 2A, miR-26a-5p significantly enhanced the expression of *IFNA*, *IFNB1*, *ISG56, IL6, CCL5*, and *CXCL10*. Consistently, Western blot analysis revealed that miR-26a-5p markedly increased the phosphorylation levels of TBK1 (p-TBK1) and IRF3 (p-IRF3) at 12 h and 24 h post-transfection (Figure 2B). In addition, ELISA analysis of cell supernatants demonstrated that miR-26a-5p significantly enhanced the secretion of IFN-α and IFN-β (Figure 2C). Given that miR-26a-5p is broadly expressed across human tissues and highly conserved across mammalian lineages [29,30], we next examined whether its immunostimulatory activity was cell-type-specific. Transfection assays were performed in human A549, U251, and HEK293T cells, as well as murine RAW264.7 and primary mouse lung cells. RT-qPCR analysis showed that miR-26a-5p significantly induced the expression of the type I interferon gene *IFNA* and the ISG *Viperin* in all tested cell types across both species (Figure 2D,E). Collectively, these results demonstrate that miR-26a-5p elicits innate antiviral immune responses, with its stimulatory activity evolutionarily conserved across multiple human and murine cell types.

### 3.2. miR-26a-5p Elicits Type I Interferon Production Independent of Its Canonical Function

**Figure 3:**
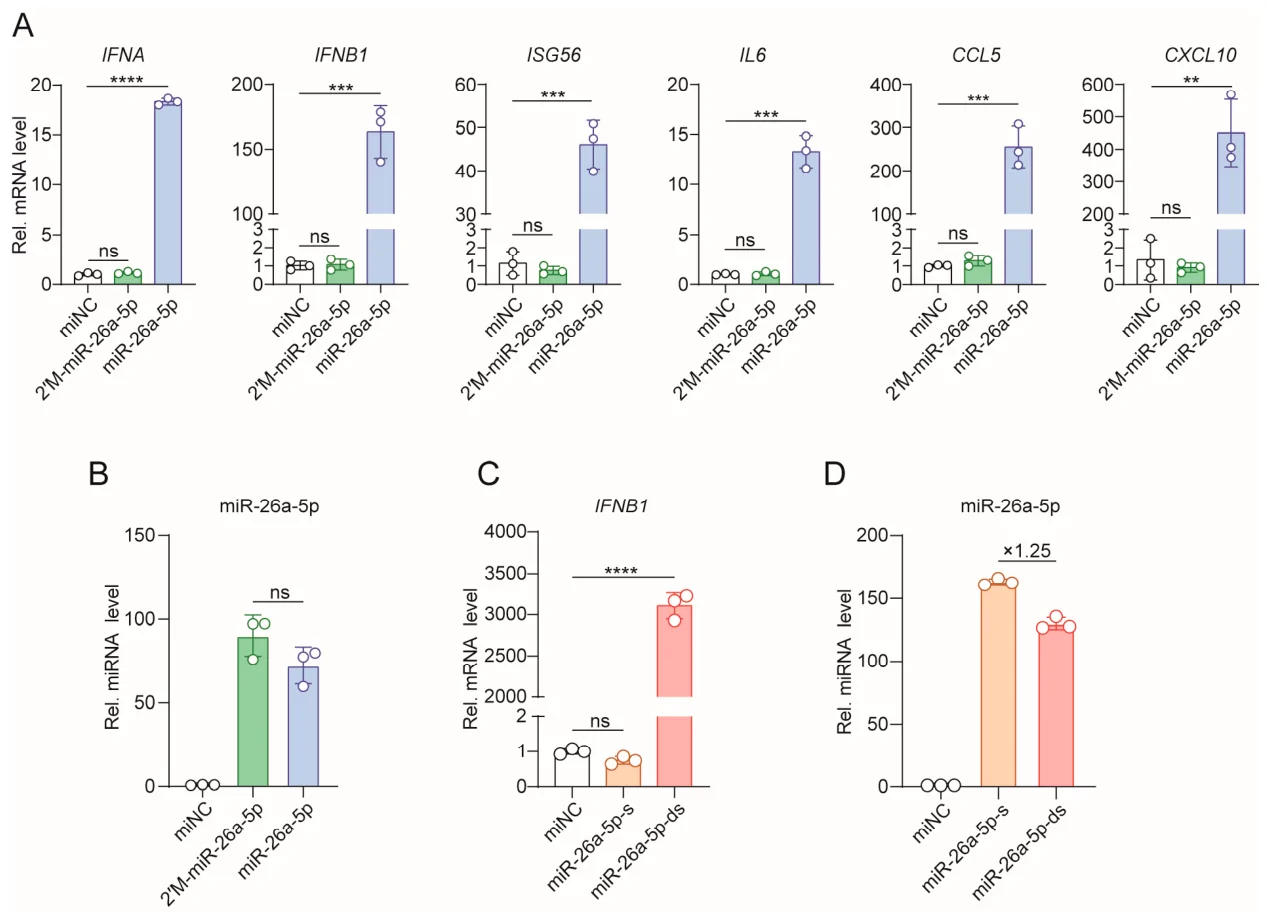
miR-26a-5p activates the type I interferon signaling pathway independently of its canonical function. (**A**) RT-qPCR analysis of the *IFNA*, *IFNB1*, *ISG56*, *IL6*, *CCL5*, and *CXCL10* mRNA levels in A549 cells 24 h post-transfection with miNC, miR-26a-5p mimic, or 2′-O-methyl-modified miR-26a-5p mimic (2′M-miR-26a-5p). (**B**) RT-qPCR analysis of intracellular miR-26a-5p levels in the same samples as described in panel A. (**C**) RT-qPCR analysis of *IFNB1* mRNA levels in A549 cells 24 h post-transfection with miNC, single-stranded miR-26a-5p mimic (miR-26a-5p-s), or double-stranded miR-26a-5p mimic (miR-26a-5p-ds). (**D**) RT-qPCR analysis of intracellular miR-26a-5p levels in the same samples as described in panel C. All miRNA mimics and their negative controls were transfected at a final concentration of 60 nM. Statistical significance was determined by Student’s *t*-test. ** *p* < 0.01, *** *p* < 0.001, **** *p* < 0.0001; ns, not significant.

Given that miRNAs canonically regulate gene expression by mediating mRNA degradation or translational repression of target transcripts, we next investigated whether miR-26a-5p induces type I interferon expression via this canonical miRNA-mediated regulatory mechanism. To distinguish its immunostimulatory effect from its conventional gene-regulatory activity, we transfected A549 cells with miNC, a double-stranded, unmodified miR-26a-5p mimic, or a double-stranded, 2′-O-methyl-modified miR-26a-5p mimic that retains target-binding capacity but abrogates RNA immunogenicity [26]. Unmodified miR-26a-5p, but not its 2′-O-methyl-modified counterpart, robustly activated the expression of *IFNA*, *IFNB1*, *ISG56*, *IL6*, *CCL5,* and *CXCL10* at 24 h post-transfection (Figure 3A,B). By contrast, transfection of a single-stranded miR-26a-5p mimic failed to induce *IFNB1* expression, yielding levels comparable to those of the negative control (Figure 3C,D). Notably, although both the 2′-O-methyl-modified and single-stranded miR-26a-5p mimics failed to trigger expression of the type I interferons, they achieved intracellular levels comparable to those of unmodified miR-26a-5p mimics upon transfection (Figure 3B,D). Together, these results demonstrate that miR-26a-5p triggers type I interferon expression independently of its canonical post-transcriptional regulatory function.

### 3.3. miR-26a-5p Induces Type I Interferon Production by Targeting RIG-I

**Figure 4:**
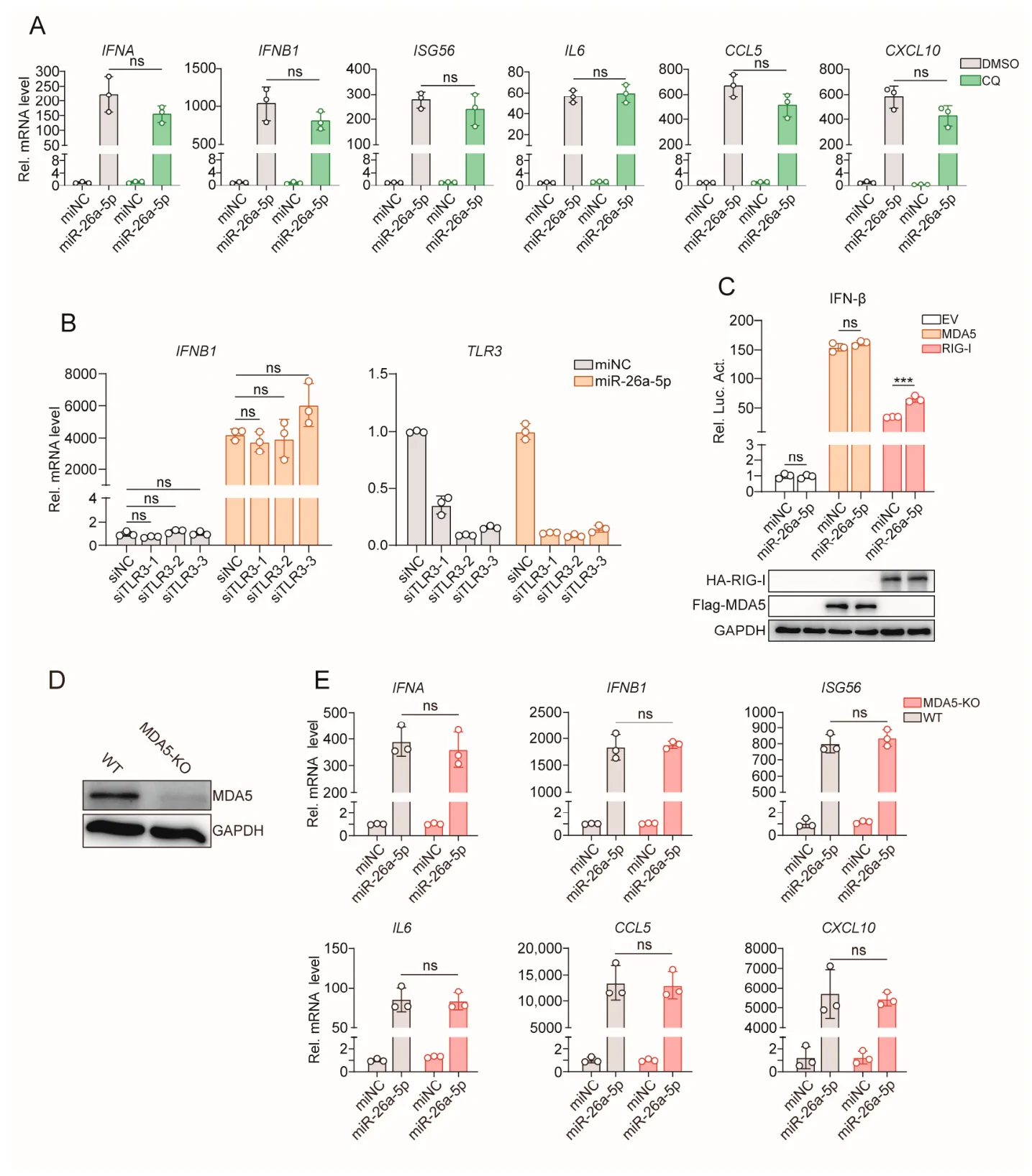
miR-26a-5p triggers type I interferon responses in a TLR- and MDA5-independent manner. (**A**) RT-qPCR analysis of *IFNA*, *IFNB1*, *ISG56*, *IL6*, *CCL5*, and *CXCL10* mRNA levels in A549 cells 24 h post-transfection with miNC or miR-26a-5p mimic, with or without a 12 h pre-treatment with 10 μM chloroquine (DMSO served as the solvent control). (**B**) RT-qPCR analysis of *IFNB1* and *TLR3* mRNA levels in A549 cells pre-transfected with siTLR3 or siNC for 36 h, followed by transfection with miNC or miR-26a-5p mimic for an additional 24 h. (**C**) Dual-luciferase reporter assay to evaluate the effect of miR-26a-5p mimic on RIG-I- or MDA5-mediated activation of the IFN-β-Luc reporter in HEK293T cells. miNC served as the negative control. (**D**) Western blot validation of MDA5 knockout in A549 MDA5-KO cells. (**E**) RT-qPCR analysis of *IFNA*, *IFNB1*, *ISG56*, *IL6*, *CCL5*, and *CXCL10* mRNA levels in A549-WT and A549 MDA5-KO cells at 24 h after transfection with miNC or miR-26a-5p mimic. miR-26a-5p mimic and its negative control were transfected at a final concentration of 60 nM, whereas siRNAs and their negative controls at 50 nM. Statistical significance was determined by Student’s *t*-test. *** *p* < 0.001; ns, not significant.

Given that miRNAs can act as ligands to directly activate TLR8 and RIG-I receptors [26,31], we hypothesized that miR-26a-5p exerts its immunostimulatory effects via an analogous mechanism. In A549 cells, intracellular RNA is known to activate type I interferon production via TLR- or RLR-dependent signaling pathways. We therefore first investigated whether TLR signaling was required for the miR-26a-5p-driven response. A549 cells were pretreated with chloroquine for 12 h to inhibit endosomal TLRs by altering endosomal and lysosomal pH [32], and then transfected with the miR-26a-5p mimic. RT-qPCR analysis revealed no significant differences in the miR-26a-5p-induced upregulation of *IFNA*, *IFNB1*, *ISG56*, *IL6*, *CCL5*, and *CXCL10* between chloroquine-treated cells and DMSO-treated control cells (Figure 4A). Since TLR7 and TLR8 recognize single-stranded RNA [33], and their basal expression is extremely low or undetectable in A549 cells [34], we further performed siRNA-mediated TLR3 knockdown to verify whether the immune-stimulatory effect of miR-26a-5p is independent of the TLR signaling pathway. Consistent with chloroquine-treatment results, siRNA-mediated TLR3 knockdown had no effect on miR-26a-5p-induced *IFNB1* expression (Figure 4B). These results demonstrate that miR-26a-5p activates type I interferon production in a TLR-independent manner.

To further investigate whether the immunostimulatory activity of miR-26a-5p relies upon RLR signaling, we performed dual-luciferase reporter assays. Cells were co-transfected with miRNA mimics, an IFN-β-Luc reporter plasmid, and overexpression plasmids encoding either RIG-I or Melanoma Differentiation-Associated Gene 5 (MDA5). Compared with control RNA, the miR-26a-5p mimic significantly enhanced RIG-I-mediated IFN-β promoter activity, yet exerted no influence over MDA5-driven reporter activity (Figure 4C). These data suggest that miR-26a-5p may specifically target RIG-I to trigger the type I interferon response. We next assessed whether MDA5 was required for miR-26a-5p-mediated type I interferon signaling via transfection assays in MDA5-knockout (MDA5-KO) A549 cells, whose knockout status was verified by Western blot analysis (Figure 4D). Wild-type A549 (A549-WT) and MDA5-KO A549 cells were separately transfected with miNC or miR-26a-5p mimic. RT-qPCR analysis showed that MDA5 deletion did not impair the immunostimulatory activity of miR-26a-5p (Figure 4E). This observation was consistent with our dual-luciferase results (Figure 4C) and indicated that miR-26a-5p operates through an MDA5-independent pathway.

**Figure 5:**
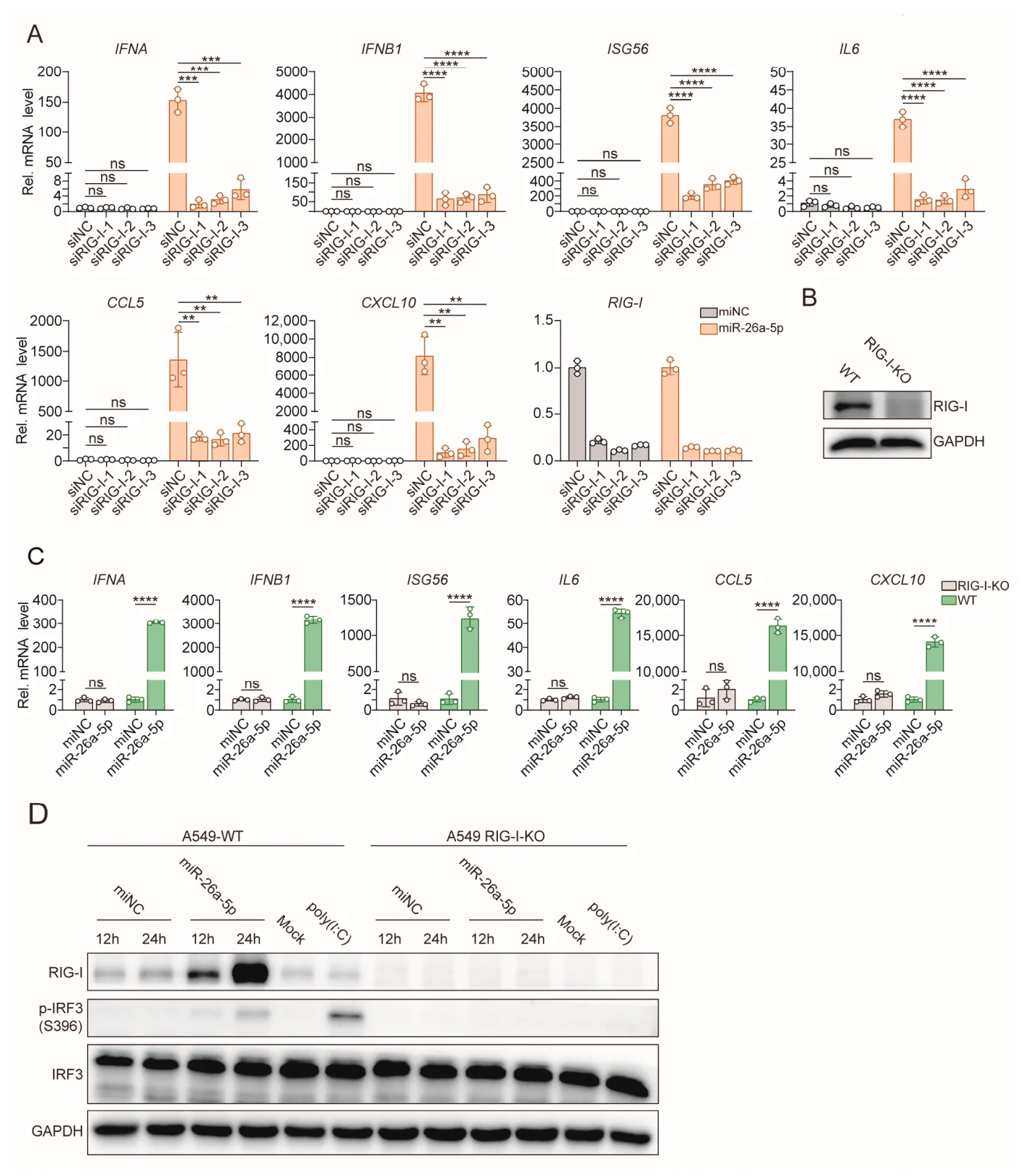
miR-26a-5p mediates activation of the type I interferon signaling pathway through targeting RIG-I. (**A**) RT-qPCR analysis of *IFNA*, *IFNB1*, *ISG56*, *IL6*, *CCL5*, *CXCL10,* and *RIG-I* mRNA levels in A549 cells pre-transfected with siRIG-I or siNC for 36 h, followed by transfection with miNC or miR-26a-5p mimic for an additional 24 h. (**B**) Western blot validation of RIG-I knockout in A549 RIG-I-KO cells. (**C**) RT-qPCR analysis of *IFNA*, *IFNB1*, *ISG56*, *IL6*, *CCL5*, and *CXCL10* mRNA levels in A549-WT and A549 RIG-I-KO cells at 24 h post-transfection with miNC or miR-26a-5p mimic. (**D**) Western blot analysis of p-IRF3 levels in A549-WT and A549 RIG-I-KO cells harvested at 12 and 24 h post-transfection with miNC or miR-26a-5p mimic. Poly(I:C) transfection for 4 h served as the positive control. miR-26a-5p mimic and its negative control were transfected at a final concentration of 60 nM; siRNAs and their negative control at 50 nM; poly(I:C) at 1 μg/mL. Statistical significance was determined by Student’s *t*-test. ** *p* < 0.01, *** *p* < 0.001, **** *p* < 0.0001; ns, not significant.

We then verified the essential role of RIG-I in this process in A549 cells using siRNA-mediated gene knockdown and CRISPR/Cas9-generated knockout cell lines. RIG-I depletion significantly attenuated the miR-26a-5p-induced upregulation of *IFNA*, *IFNB1*, *ISG56*, *IL6*, *CCL5*, and *CXCL10* (Figure 5A). In contrast, RIG-I knockout completely abrogated these stimulatory effects (Figure 5B,C). To confirm that RIG-I is required for miR-26a-5p-mediated signaling at the protein level, A549-WT and RIG-I-knockout A549 (A549 RIG-I-KO) cells were transfected with miNC, miR-26a-5p mimic, or poly(I:C). As seen in Figure 5D, the miR-26a-5p mimic significantly increased p-IRF3 protein levels relative to controls in A549-WT cells at 12 and 24 h post-transfection. In contrast, p-IRF3 was undetectable in A549 RIG-I-KO cells in both the miR-26a-5p treated and control groups. These results indicate that miR-26a-5p elicits type I interferon signaling in a strictly RIG-I-dependent manner. Collectively, these results demonstrate that miR-26a-5p activates type I interferon signaling by specifically targeting RIG-I.

### 3.4. miR-26a-5p Acts as a Ligand That Binds and Activates RIG-I

**Figure 6:**
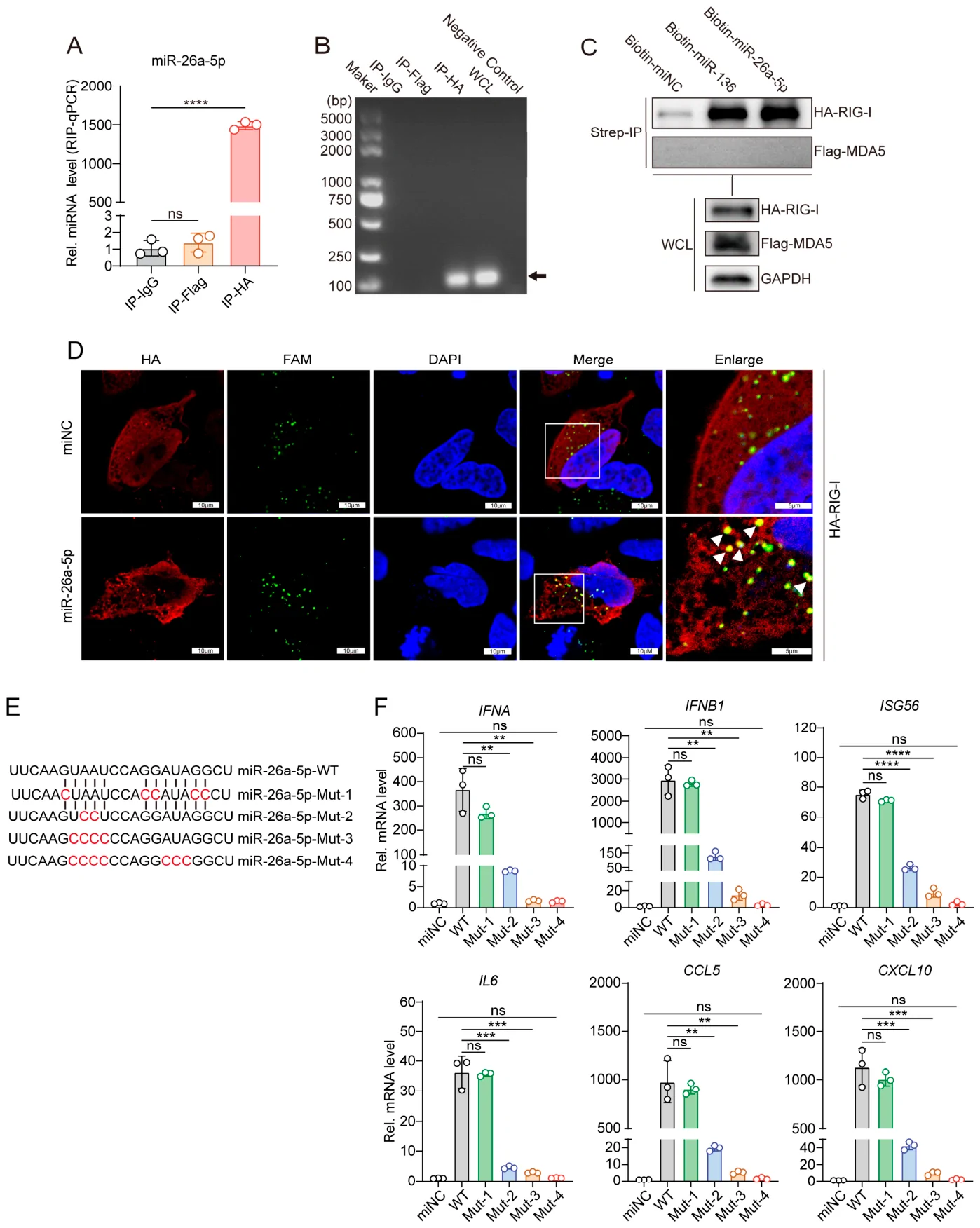
Interaction and intracellular colocalization of miR-26a-5p with RIG-I. (**A**) RIP assays validate the interaction between miR-26a-5p and RIG-I. HEK293T cells were separately transfected with HA-RIG-I or Flag-MDA5 expression plasmids, followed by transfection of the miR-26a-5p mimic at 24 h post-transfection. At 12 h after the secondary transfection, RIP assays were performed using anti-HA or anti-Flag antibody, with the corresponding IgG antibody included as a negative control. RT-qPCR was applied to quantify miR-26a-5p enrichment in RNA extracted from immunoprecipitated complexes, and the miR-26a-5p enrichment level was normalized to the IgG isotype control. (**B**) Conventional RT-PCR detection of miR-26a-5p in the same RIP-eluted RNA samples as described in panel A. The black arrow marks the specific miR-26a-5p band amplified by PCR following stem-loop reverse transcription. (**C**) RNA pull-down assays validate the interaction between miR-26a-5p and RIG-I. HEK293T cells were separately transfected with HA-RIG-I or Flag-MDA5 expression plasmids. At 24 h post-transfection, biotin-labeled miNC, miR-136 mimic, and miR-26a-5p mimic were used as baits to pull down RNA-binding protein complexes. Enrichment of RIG-I and MDA5 proteins was detected by Western blot analysis. (**D**) Confocal immunofluorescence microscopy analysis of the intracellular colocalization between FAM-labeled miNC or miR-26a-5p mimic and HA-tagged RIG-I in A549 cells. White arrowheads indicate the colocalization of FAM-labeled miR-26a-5p mimic and HA-tagged RIG-I; white squares indicate the regions shown in the enlarged views. (**E**) Sequences of wild-type and multiple mutant miR-26a-5p mimics. (**F**) RT-qPCR analysis of *IFNA*, *IFNB1*, *ISG56*, *IL6*, *CCL5*, and *CXCL10* mRNA levels in A549 cells at 24 h post-transfection with miNC, wild-type miR-26a-5p mimic, and multiple mutant miR-26a-5p mimics. All miRNA mimics and their corresponding negative controls were transfected at a final concentration of 60 nM. Statistical significance was determined by Student’s *t*-test. ** *p* < 0.01, *** *p* < 0.001, **** *p* < 0.0001; ns, not significant.

Given that miR-26a-5p was co-immunoprecipitated with RIG-I in our RIP-seq screen, we next investigated whether it engages RIG-I in a manner analogous to the known RIG-I ligand miR-136. To this end, we performed RIP-qPCR assays in HEK293T cells transfected with HA-RIG-I or Flag-MDA5 overexpression plasmids, followed by transfection with the miR-26a-5p mimic 24 h later. RIP assays were performed 12 h later using anti-HA or anti-Flag antibodies together with their respective isotype-matched IgG controls. RT-qPCR analysis revealed that miR-26a-5p was significantly enriched in RIG-I precipitates, but not in MDA5 precipitates, relative to IgG isotype controls (Figure 6A). This selective enrichment was further validated by conventional RT-PCR analysis (Figure 6B). To further confirm the direct interaction, reciprocal biotin-labeled RNA pull-down assays were performed. HEK293T cells were transfected with HA-RIG-I or Flag-MDA5 overexpression plasmids. At 24 h post-transfection, RNA pull-down assays were conducted using biotin-labeled miNC (negative control), miR-136 (positive control), and miR-26a-5p as baits. As shown in Figure 6C, compared with miNC, RIG-I but not MDA5 was specifically pulled down by biotin-labeled miR-26a-5p, consistent with the positive control miR-136. Collectively, the RIP and reciprocal RNA pull-down assays demonstrate a direct physical interaction between miR-26a-5p and RIG-I. We next examined their intracellular colocalization. A549 cells co-transfected with the HA-RIG-I overexpression plasmid and either FAM-labeled miR-26a-5p mimic or FAM-labeled miNC were subjected to immunofluorescence staining for the HA tag. As shown in Figure 6D, prominent colocalization of FAM-labeled miR-26a-5p with HA-RIG-I was observed in the cytoplasm, whereas no such colocalization was detected in the miNC control group. Taken together, these results demonstrate that miR-26a-5p serves as a ligand that directly binds to and activates RIG-I, thereby triggering the type I interferon signaling pathway.

RIG-I is known to preferentially recognize dsRNA bearing 5′-ppp or 5′-pp moieties, with 5′-terminal polyphosphates serving as the core molecular determinant governing RIG-I recognition and activation [5]. Additionally, emerging evidence indicates that the internal sequences of an RNA ligand potently modulate both RIG-I binding affinity and the magnitude of downstream signaling activation, representing a critical regulatory layer in this process [35,36]. AU-rich and poly(U/UC) motifs have been validated as key RNA determinants driving RIG-I recognition and activation [35,37,38]. To determine whether AU-rich sequences also serve as a critical sequence feature in miR-26a-5p-mediated RIG-I activation, we generated a series of miR-26a-5p point mutants (Figure 6E). Relative to the wild-type miR-26a-5p mimic, mutating the UAAU motif to UCCU (Mut-2) reduced *IFNB1* mRNA induction to 4.35% of the wild-type level, while a UAAU-to-CCCC substitution (Mut-3) reduced it to only 0.51% (Figure 6F). Moreover, substituting all AU-rich sequences with C residues (Mut-4) completely abrogated RIG-I activation. In contrast, G-to-C substitutions (Mut-1), which left the AU-rich motifs intact and thus served as a control to rule out non-specific mutational effects, had no significant impact on *IFNB1* induction (Figure 6F). Collectively, these mutagenesis data identify AU-rich motifs as key sequence determinants governing miR-26a-5p-triggered RIG-I activation.

### 3.5. miR-26a-5p Exerts Broad-Spectrum Antiviral Activity by Activating RIG-I

**Figure 7:**
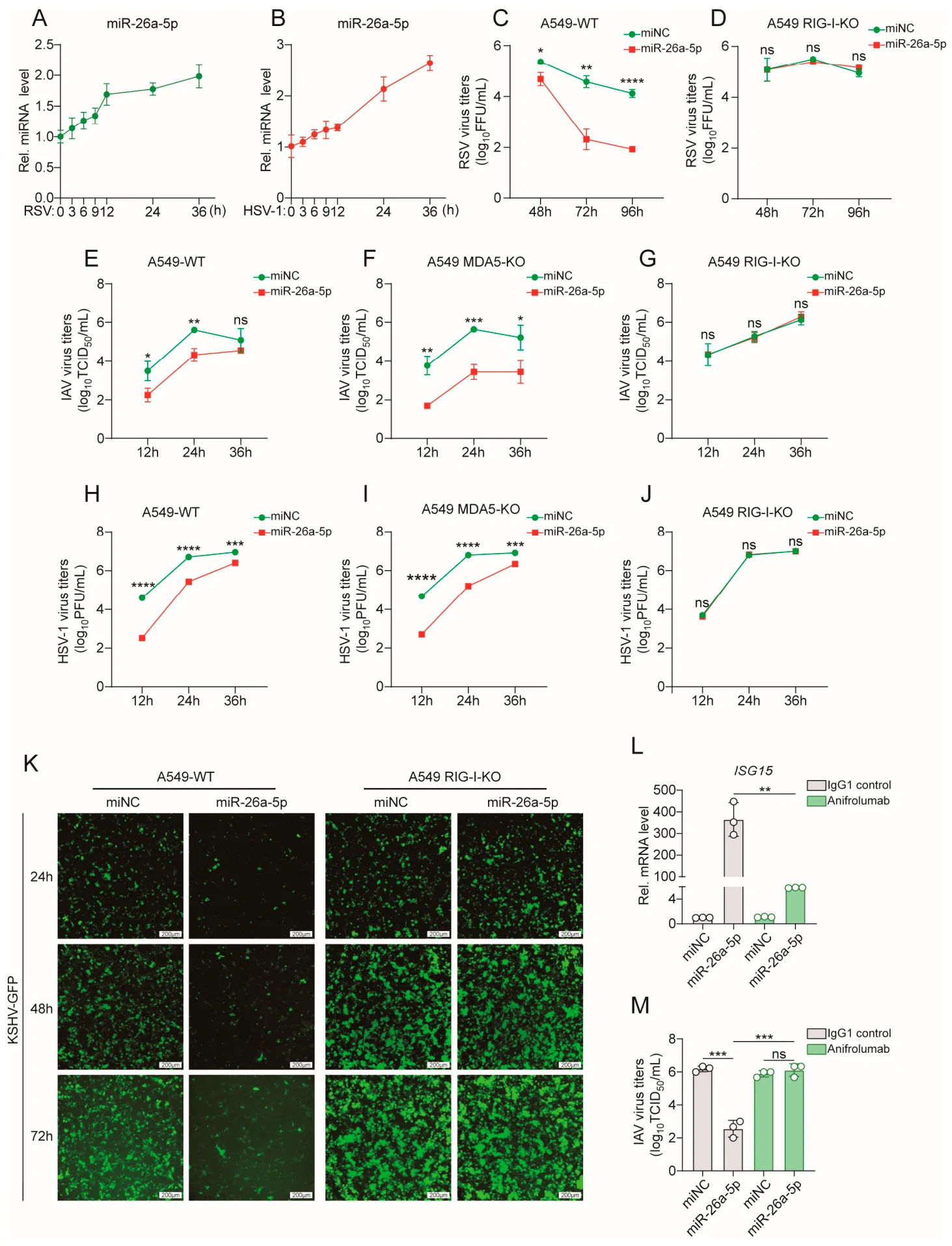
miR-26a-5p exerts RIG-I-dependent broad-spectrum antiviral activity against both RNA and DNA viruses. (**A**,**B**) Expression of miR-26a-5p in A549 cells infected with RSV (**A**) or HSV-1 (**B**) at an MOI of 1 at the indicated time points. (**C**–**K**) A549-WT, A549 MDA5-KO, and A549 RIG-I-KO cells were first transfected with miNC or miR-26a-5p mimic at a final concentration of 60 nM. Twenty-four hours after transfection, cells were infected with RSV, IAV, HSV-1, and KSHV-GFP to evaluate the regulatory effect of miR-26a-5p on viral replication. Shown are the effects of miR-26a-5p on RSV replication in A549-WT (**C**) and A549 RIG-I-KO cells (**D**); IAV (PR8 strain) replication in A549-WT (**E**), A549 MDA5-KO (**F**), and A549 RIG-I-KO cells (**G**); HSV-1 replication in A549-WT (**H**), A549 MDA5-KO (**I**), and A549 RIG-I-KO cells (**J**); and KSHV-GFP replication in A549-WT and A549 RIG-I-KO cells (**K**). (**L**) Anifrolumab effectively blocks miR-26a-5p-induced type-I-IFN-dependent ISG15 expression. (**M**) The antiviral effects of miR-26a-5p against IAV are mitigated by Anifrolumab-mediated inhibition of type-I IFN signaling. Statistical significance was assessed by Student’s *t*-test. * *p* < 0.05, ** *p* < 0.01, *** *p* < 0.001, **** *p* < 0.0001; ns, not significant.

Given that type I interferons are central to host antiviral defense, our finding that miR-26a-5p acts as a RIG-I ligand and potently induces type I interferon production suggests that this miRNA may have substantial antiviral potential. To explore its functional relevance during viral infection, we measured miR-26a-5p expression following infection with RSV (an RNA virus) and HSV-1 (a DNA virus). miR-26a-5p was moderately upregulated upon infection with both RSV and HSV-1 (Figure 7A,B), hinting at its potential function in antiviral immunity against diverse viruses. We first evaluated the antiviral activity of miR-26a-5p against RNA viruses. A549-WT and A549 RIG-I-KO cells were transfected with miNC or miR-26a-5p mimic and then infected with RSV at 24 h post-transfection. In A549-WT cells, miR-26a-5p significantly reduced viral titers relative to the miNC control; this effect was abolished in A549 RIG-I-KO cells (Figure 7C,D). Similarly, miR-26a-5p exhibited potent activity against IAV (PR8 strain) in A549-WT and A549 MDA5-KO cells, whereas RIG-I deletion completely eliminated this protection (Figure 7E–G). Together, these data demonstrate that miR-26a-5p exerts RIG-I-dependent antiviral activity against RNA viruses.

We next examined whether miR-26a-5p also exerts antiviral activity against DNA viruses. In HSV-1-infected A549-WT and A549 MDA5-KO cells, miR-26a-5p significantly inhibit viral replication compared with the miNC control; in A549 RIG-I-KO cells, however, this inhibitory effect was lost (Figure 7H–J). We extended these observations to a recombinant GFP-expressing KSHV-GFP [39]. Consistent with the HSV-1 results, miR-26a-5p markedly suppressed KSHV-GFP replication in A549-WT cells, as measured by GFP fluorescence, but had no effect in A549 RIG-I-KO cells (Figure 7K). Notably, because miR-26a-5p itself serves as a RIG-I agonist, the resulting type I interferon response confers protection irrespective of the genome type of the infecting virus; this explains why RIG-I—a canonical RNA sensor—is required for miR-26a-5p-mediated antiviral activity even against DNA viruses. To confirm this, we carried out validation assays with the IFNAR1-blocking antibody Anifrolumab under IAV-infection conditions. Compared with the IgG control group, Anifrolumab significantly inhibited the antiviral activity of miR-26a-5p (Figure 7L,M). Collectively, these results demonstrate that miR-26a-5p exerts broad-spectrum antiviral activity against both RNA and DNA viruses, and this protective effect is predominantly driven by miR-26a-5p-mediated RIG-I activation, which subsequently initiates type I interferon signaling.

## 4. Discussion

RIG-I is a core mediator of host antiviral innate immunity against RNA viruses and a subset of DNA viruses. Its activation state is tightly controlled by a broad array of host factors, among which miRNAs have attracted increasing attention [5]. To date, however, only a very limited number of miRNAs have been reported to act as ligands that bind and activate RIG-I [26]; such non-canonical, immune-activating miRNAs remain rare, and the architecture of the underlying regulatory network is still poorly defined. Here, we identify miR-26a-5p as a previously unrecognized ligand of RIG-I. miR-26a-5p directly binds to and activates RIG-I, initiating RLR signaling and promoting type I interferon production. This activity confers broad-spectrum protection against both RNA and DNA viruses.

Since the discovery of RIG-I, pathogen-derived RNAs from invading viruses have long been regarded as its primary agonists. However, accumulating evidence has revealed that host-derived RNAs, including RNA5SP141, vtRNAs, Y-RNAs, and ITS2, can also serve as ligands that bind to and activate RIG-I during viral infection [5,11,40]. miRNAs generally function via canonical post-transcriptional regulatory mechanisms, and most reported effects of miRNAs on the RLR pathway rely on such mechanisms [25,41], whereas miR-136 was reported to activate RIG-I via a non-canonical function [26]. We therefore investigated whether the type I interferon-inducing activity of miR-26a-5p likewise operates independently of canonical miRNA function. Assays using 2′-O-methyl-modified and single-stranded miR-26a-5p mimics demonstrated that miR-26a-5p-induced type I interferon production is independent of its canonical function. Because miR-26a-5p is expressed across many human tissues and is highly conserved among mammals [29,30], we further validated its immune-activating activity in a panel of human and murine cell lines. The consistent induction of type I interferon production across these diverse cellular contexts demonstrates that this regulatory activity is functionally conserved across species.

Intracellular RNA-induced type I interferon signaling is mainly governed by two receptor systems: the endosomal TLRs and the cytoplasmic RLRs [42]. Because host-encoded miR-21 and miR-29a have been shown to bind and activate human TLR8 [31], we first investigated whether miR-26a-5p exerts its immunostimulatory activity through the TLR signaling cascade. Treatment with chloroquine, an inhibitor of endosomal acidification that blocks endosomal-TLR activation [32], did not impair miR-26a-5p-mediated activation of the type I interferon signaling pathway, indicating that miR-26a-5p functions independently of TLR signaling. This notion was further supported by siRNA-mediated knockdown of TLR3. Having excluded TLR involvement, we next focused on the cytoplasmic RLRs, which serve as the primary sensors of cytosolic RNA and initiate type I interferon responses, to identify the direct target of miR-26a-5p. The mammalian RLR family includes two sensors with distinct and non-redundant ligand specificities: RIG-I, which preferentially recognizes short RNAs bearing 5′-triphosphate moieties or compact stem-loop structures, and MDA5, which is selectively activated by long dsRNA duplexes [43]. Dual-luciferase reporter assays demonstrated that miR-26a-5p specifically potentiated RIG-I-mediated signaling, without enhancing MDA5-driven signaling. This selectivity was further confirmed by genetic loss-of-function experiments: transient knockdown of RIG-I markedly attenuated miR-26a-5p-triggered innate antiviral immune responses, and complete RIG-I ablation abolished these immunostimulatory effects, whereas MDA5 deficiency had no effect. Thus, miR-26a-5p functions specifically through RIG-I rather than MDA5, establishing RIG-I as the essential, non-redundant mediator of miR-26a-5p-driven innate immune activation. To confirm that miR-26a-5p acts as a RIG-I agonist, analogous to our previous findings for miR-136 [26], we performed a series of orthogonal biochemical and confocal imaging assays. RIP and RNA pull-down assays revealed a robust direct interaction between miR-26a-5p and RIG-I protein, and confocal immunofluorescence confirmed their prominent co-localization in the cytoplasm, the physiological compartment of RIG-I-mediated RNA sensing. Together, these findings define a novel TLR-independent regulatory axis in innate immunity, wherein miR-26a-5p acts as a RIG-I-specific ligand to trigger type I interferon signaling. Importantly, this broadens the functional repertoire of mammalian miRNAs beyond canonical post-transcriptional gene silencing and identifies miR-26a-5p as a new RIG-I agonist.

Although 5′-triphosphate modification of PAMP RNA is a prerequisite for RIG-I engagement, it alone is not sufficient for stable binding, which also depends on homopolymeric ribonucleotide composition, linear structure, and RNA length [35]. In hepatitis C virus (HCV) infection, the A/U content and poly(U/UC) motifs are key determinants of RIG-I recognition of viral PAMP RNA [35,36]. A similar sequence-dependent recognition pattern is observed in measles virus infection, where RIG-I preferentially binds AU-rich viral RNA sequences and higher AU content confers stronger immunostimulatory activity [38]. Consistent with this, our targeted mutagenesis identified the UAAU motif as a critical determinant of miR-26a-5p-mediated RIG-I activation, and comprehensive mutation of all AU-rich elements completely abolished its immunostimulatory capacity. These results establish AU-rich sequences as pivotal functional determinants of miR-26a-5p-driven RIG-I activation. In addition, the three-dimensional conformation of miR-26a-5p, shaped collectively by its AU-rich sequences and flanking nucleotide regions, may also partially contribute to miR-26a-5p-mediated RIG-I activation.

miR-26a-5p exerts antiviral functions via the canonical miRNA mode of action. On one hand, it directly targets and degrades viral RNA to suppress SARS-CoV-2 replication [44]. On the other hand, it downregulates the expression of USP15, USP3, and SOCS5—negative regulators of type I interferon signaling—thereby enhancing virus-induced type I interferon responses and restricting the replication of the DNA virus FHV-1, as well as multiple RNA viruses, including HEV, VSV, SeV, and IAV [45,46,47]. Other studies have revealed that miR-26a-5p inhibits PRRSV replication by inducing type I interferon production but did not identify the upstream PRR, leaving a key mechanistic gap [48,49]. Building on these findings, our study broadens the antiviral spectrum of miR-26a-5p, showing that it significantly suppresses both RNA viruses (RSV and IAV) and DNA viruses (HSV-1 and KSHV-GFP) in A549 cells, in agreement with previous reports [45,46]. Most importantly, the antiviral effect of miR-26a-5p was completely abrogated in A549 RIG-I-KO cells but not in A549 MDA5-KO cells, indicating that the broad-spectrum antiviral activity is strictly dependent on RIG-I, which is inconsistent with our earlier observations that RIG-I is the obligate, non-redundant mediator of miR-26a-5p’s immune-activating function. We therefore propose an antiviral mechanism distinct from the canonical mode: rather than inhibiting viral replication by targeting viral RNA or host factors, miR-26a-5p acts as a ligand that binds and activates RIG-I via a non-canonical function. This activation triggers the type I interferon response and ultimately exerts broad-spectrum antiviral activity. This non-canonical function complements the previously reported canonical regulatory mechanisms, and together they constitute the complete antiviral regulatory network of miR-26a-5p.

Nevertheless, several limitations of the present study should be acknowledged. All functional experiments were performed via exogenous overexpression of the miR-26a-5p mimic. No loss-of-function experiments were carried out to verify the biological function of endogenous miR-26a-5p. Consequently, we cannot completely rule out the possibility that the observed phenotypes may partially arise from non-physiological effects caused by supraphysiological levels of exogenous miR-26a-5p. Future studies employing genetic knockout models of miR-26a-5p are warranted to further define the immunomodulatory role of endogenous miR-26a-5p in the context of viral infection.

In summary, our study reveals a previously unrecognized non-canonical function of miR-26a-5p in orchestrating antiviral innate immunity. Mechanistically, miR-26a-5p acts as a ligand that binds and activates RIG-I, initiating the type I interferon signaling cascade and mediating potent broad-spectrum antiviral activity against a wide range of RNA and DNA viruses. This work extends the known pool of RIG-I agonists and advances our understanding of non-canonical miRNA-mediated innate immune regulation. Furthermore, these findings offer insights for developing broad-spectrum immunomodulatory approaches to combat viral infections.

## Data Availability

The raw RIP-seq datasets generated in this study have been deposited in the Science Data Bank (ScienceDB) and are publicly accessible under the accession DOI: https://doi.org/10.57760/sciencedb.36277.

## Supplementary Materials

The following supporting information can be downloaded at: https://www.mdpi.com/article/10.3390/v18091005/s1, Supplementary Table S1. The qPCR primer sequences used in this study. Supplementary Table S2. miRNAs identified via RIG-I-baited RIP-seq.
