# Supplementary material for: miR-26a-5p Activates Antiviral Innate Immunity via Direct Binding and Activation of RIG-I
Source: Viruses. 2026 Sep 12;18(9):1005. doi: 10.3390/v18091005 (PMC13612082; doi:10.3390/v18091005)
Supplement: Supplementary file 1 [file viruses-18-01005-s001.zip › Supplementary Table S1.pdf]

Supplementary Table S1. The qPCR primers sequence used in this study

Human Gene		
**Gene**	**Forward (5′-3′)**	**Reverse (5′-3′)**
IFNA	GTTCCAGAAGGCTCAAGCCATC	TAGGAGGCTCTGTTCCCAAGCA
IFNB1	CTTGGATTCCTACAAAGAAGCAGC	TCCTCCTTCTGGAACTGCTGCA
ISG56	GCCTTGCTGAAGTGTGGAGGAA	ATCCAGGCGATAGGCAGAGATC
CCL5	CCTGCTGCTTTGCCTACATTGC	ACACACTTGGCGGTTCTTTCGG
CXCL10	GGTGAGAAGAGATGTCTGAATCC	GTCCATCCTTGGAAGCACTGCA
RIG-I	CACCTCAGTTGCTGATGAAGGC	GTCAGAAGGAAGCACTTGCTACC
ACTB	GCCGGGACCTGACTGACTAC	CGGATGTCCACGTCACACTT
IL6	AGACAGCCACTCACCTCTTCAG	TTCTGCCAGTGCCTCTTTGCTG
Viperin	CCAGTGCAACTACAAATGCGGC	CGGTCTTGAAGAAATGGCTCTCC
ISG15	CGCAGATCACCCAGAAGATCG	TTCGTCGCATTTGTCCACCA
TLR3	GCGCTAAAAAGTGAAGAACTGGAT	GCTGGACATTGTTCAGAAAGAGG
**Mouse Gene**		
Viperin	TGCTGGCTGAGAATAGCATTAGG	GCTGAGTGCTGTTCCCATCT
Ifnα	CAGCTCCAAGAAAGGACGAAC	GGCAGTGTAACTCTTCTGCAT
Actb	ACGGCCAGGTCATCACTATT	TGGCATAGAGGTCTTTACGGA
